# Patient-reported outcomes in urticarial vasculitis treated with omalizumab: case report

**DOI:** 10.1186/s12895-018-0077-x

**Published:** 2018-10-25

**Authors:** Ivan Cherrez-Ojeda, Emanuel Vanegas, Miguel Felix, Valeria L. Mata, Annia Cherrez

**Affiliations:** 1 Universidad Espíritu Santo, Km. 2.5 vía La Puntilla, 0901-952, Samborondon, Guayaquil Ecuador; 2 Respiralab, Respiralab Research Group, Guayaquil, Ecuador; 3 Dermatology Department, University Hospital, Rostock, Germany

**Keywords:** Urticarial vasculitis, Patient-reported outcomes, Omalizumab

## Abstract

**Background:**

Despite the current knowledge of UV, there is a lack of consensus among diagnostic criteria and management. In general, antihistamine therapy is regularly used for the symptomatic management of pruritus but does not control inflammation or alter the course of the disease. Monoclonal antibodies such as omalizumab (anti-IgE) have been proposed as a potential treatment for urticarial vasculitis. A few studies have reported the benefits of omalizumab in patient-reported outcome measures (PROMs). Herein we describe a female patient with urticarial vasculitis who was treated with omalizumab. We discuss the response to treatment and possible implications of PROMs in guiding the management of the disease.

**Case presentation:**

We describe the case of a 57-year-old woman with a diagnosis of urticarial vasculitis. Due to lack of response to first-line treatment and the severity of the disease, treatment with omalizumab was initiated. Omalizumab 150 mg was administered every four weeks for three months. Second-generation antihistamines were used as needed. Both CU-Q2oL and UAS 7 improved. After three-month therapy with omalizumab, disease severity improved from moderate severity (UAS7 = 19) to well controlled (UAS7 = 6). However, 5 months after the last administration of omalizumab, the patient complained of worsening symptoms and active disease with quality of life impairment. A single dose of omalizumab (150 mg) was prescribed with corticosteroids. Thereafter, the patient presented a disease activity and quality of life with a fluctuating pattern that was controlled with additional doses of omalizumab.

**Conclusion:**

In chronic urticaria, patient-reported outcome measures (PROMs) are important for assessing disease status and the impact of symptoms on patients’ lives. However, to our knowledge, there is no validated tool to measure such outcomes in UV patients. Although UAS7 and CU-Q2oL were not designed for UV assessment, they might be useful in the clinical setting as objective measures to determine treatment efficacy. However, some domains in the CU-Q2oL questionnaires do not correlate well with UAS7, which might serve as a relative indication to continue treatment despite disease severity improvement. Based on our observations, we believe omalizumab 150 mg might be a feasible therapeutic alternative when first-line treatment is unsuccessful.

## Background

Urticarial vasculitis (UV) is a clinicopathological entity consisting of clinical manifestations of urticaria and histopathological evidence of small vessel cutaneous leukocytoclastic vasculitis (LCV) [1]. Clinically, lesions typically persist beyond 24 h, often resolving with faint residual hyperpigmentation. Vasculitic lesions can be pruritic in nature, but more commonly present in an asymptomatic or painful way (often with a stinging or burning sensation) [2]. Histopathological lesions consist of an inflammatory manifestation with injury to the capillaries and postcapillary venules in the skin [3]. Leukocytoclasis and fibrinoid deposits appear to be the most distinguishing features of LCV and are direct signs of vessel damage [4].

UV is a relatively uncommon disease, with a prevalence ranging from 2 to 20% among chronic urticaria patients (CU) [5]. In a previous study, we found the prevalence to be approximately 10% of CU patients [6]. It is more common among women, with a peak incidence around the fourth decade of life [5]. Regarding the etiology, most cases appear to be idiopathic. UV can also be associated with connective-tissue diseases, particularly systemic lupus erythematosus (SLE) and Sjogren’s syndrome [7]. Malignancies, chronic infections, serum sickness, drugs, and sun exposure are also associated with UV [7]. Systemic manifestations of UV can include constitutional symptoms, musculoskeletal, renal, ophthalmic, pulmonary, gastrointestinal, neurologic, and even cardiovascular involvement [8].

Serum complement levels are of particular importance. Patients with low complement levels usually present more systemic involvement, while normocomplementemic patients have a milder course [9]. Among the recognized syndromes of low complement levels in association with UV, are hypocomplementemic urticarial vasculitis syndrome (HUVS), and hypocomplementemic urticarial vasculitis (HUV) [5]. HUVS, also known as McDuffie syndrome, is recognized as an autoimmune disorder with at least 6 or more months of urticaria in the presence of hypocomplementemia, and various systemic manifestations (including arthritis, arthralgias, glomerulonephritis, uveitis, episcleritis, and recurrent abdominal pain) [10]. On the other hand, HUV are patients who do not meet criteria for HUVS, but still present with low complement levels. In comparison to HUVS, HUV patients present with fewer systemic manifestations [5].

Despite the current knowledge of UV, there is a lack of consensus among diagnostic criteria and management. Treatment varies from patient to patient according to the disease severity and clinical presentation. In general, antihistamine therapy is regularly used for the symptomatic management of pruritus but does not control inflammation or alter the course of the disease [11]. Hydroxychloroquine (HCQ) appears to be as effective as corticosteroids among first-line therapy options [8]. The immunosuppressive agents azathioprine (AZA), mycophenolate mofetil (MMF), rituximab, or cyclophosphamide may be used in patients with relapsing or refractory disease [8]. Dapsone, colchicine, and cyclosporine have been used as therapeutic alternatives, mostly with unsatisfying results [12].

Monoclonal antibodies such as omalizumab (anti-IgE) have been proposed as a potential treatment for urticarial vasculitis [13]. A few studies have reported the benefits of omalizumab in patient-reported outcome measures (PROMs) [14]. Herein we describe a female patient with urticarial vasculitis who was treated with omalizumab. We discuss the response to treatment and possible implications of PROMs in guiding the management of the disease.

## Case presentation

**Fig. 1:**
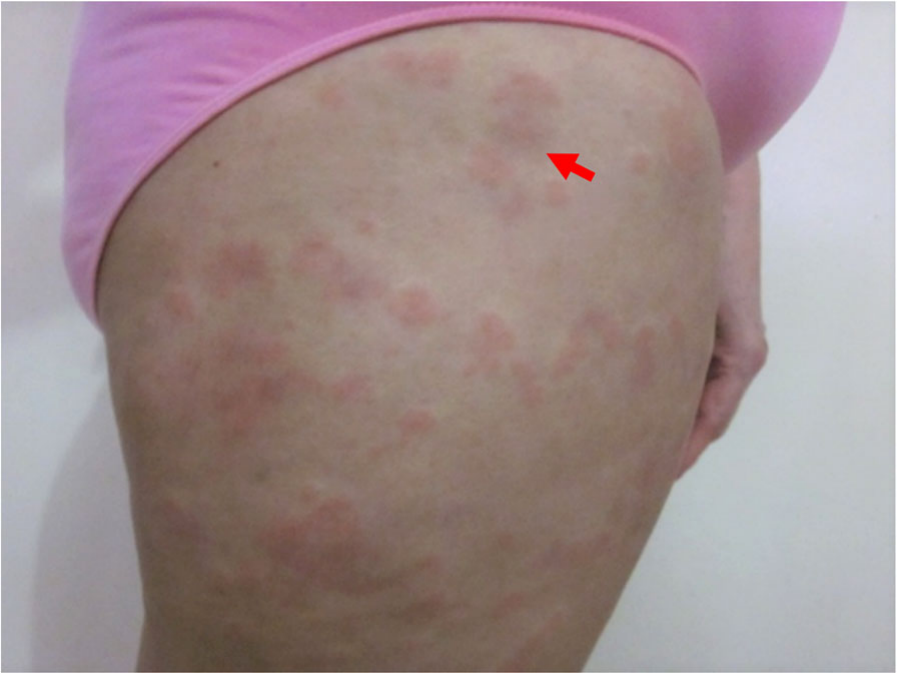
Urticarial vasculitis lesions on lateral portion of right thigh. Clinical characteristics of lesions included generalized burning sensation of the skin, with plaques persisting for more than 24 h and leaving faint residual hyperpigmentation of the skin (highlighted by the arrow)

**Fig. 2:**
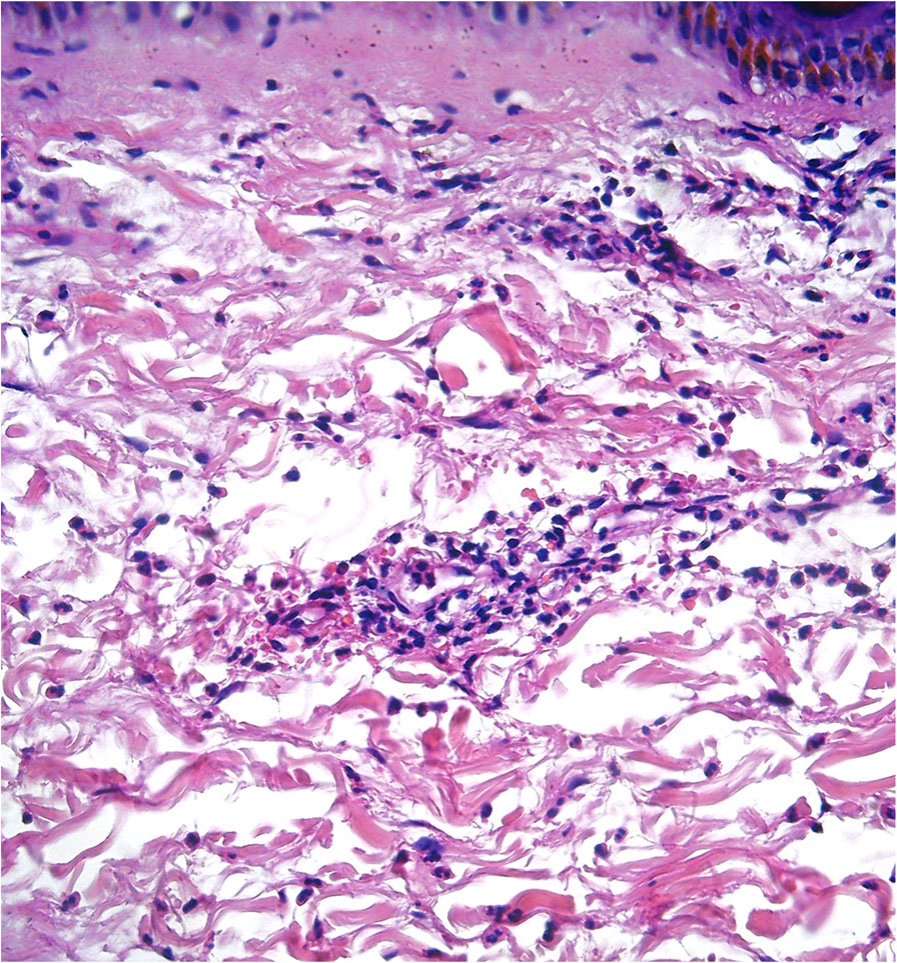
Histopathology of urticarial vasculitis lesions showing leukocytoclastic vasculitis with perivascular infiltrates and neutrophil predominance

A 57-year-old woman presented to our office with complaints of wheals, arthralgias, and a severe, generalized burning sensation on the skin. The skin lesions appeared as urticarial plaques located mainly on the trunk and proximal extremities persisting for more than 24 h after the initial appearance and leaving faint residual hyperpigmentation on the skin (Fig. 1). The patient reported approximately 6 months of relapsing and remitting symptoms. No angioedema or relevant past medical history were noted. On the basis of the previous findings, a possible diagnosis of urticarial vasculitis was suspected, and laboratory tests with a skin biopsy were requested. Laboratory tests were unremarkable; no abnormalities were noted on hemogram, acute phase reactants, thyroid function, or complement levels. Skin biopsy revealed leukocytoclastic vasculitis with perivascular infiltrates and neutrophil predominance (Fig. 2). After a careful assessment, the patient was diagnosed with normocomplementemic urticarial vasculitis.

The patient was initially treated with a short course of oral corticosteroids (prednisone 40 mg initially for 4 days, followed by gradual tapering off for a total of 12 days), first generation H1 antihistamine (hydroxyzine 50 mg taken at night), second generation H1 antihistamine (fexofenadine 20 mg up to fourfold dosage), and hydroxychloroquine 200 mg daily for 4 months. Despite the initial treatment, the symptoms did not improve, and the UV appeared as a more active and severe disease during physical examination. In addition, the patient was disappointed with how her quality of life was markedly impaired due to her clinical condition. For this reason, Urticaria Activity Score 7 (UAS7) and Chronic Urticaria Quality of Life Questionnaire (CU-Q2oL) were assessed for the first time to have a more objective course of the disease. Her UAS7 immediately after the 4-month course of first-line therapy was 19, while CU-Q2oL showed a functioning status of 29.2, sleep of 12.5, itching/embarrassment of 25.0, mental status of 25.0, swelling/eating of 18.8, and limited appearance of 25.0. Due to the lack of response to first-line treatment and the severity of the disease, treatment with omalizumab was initiated.

**Fig. 3:**
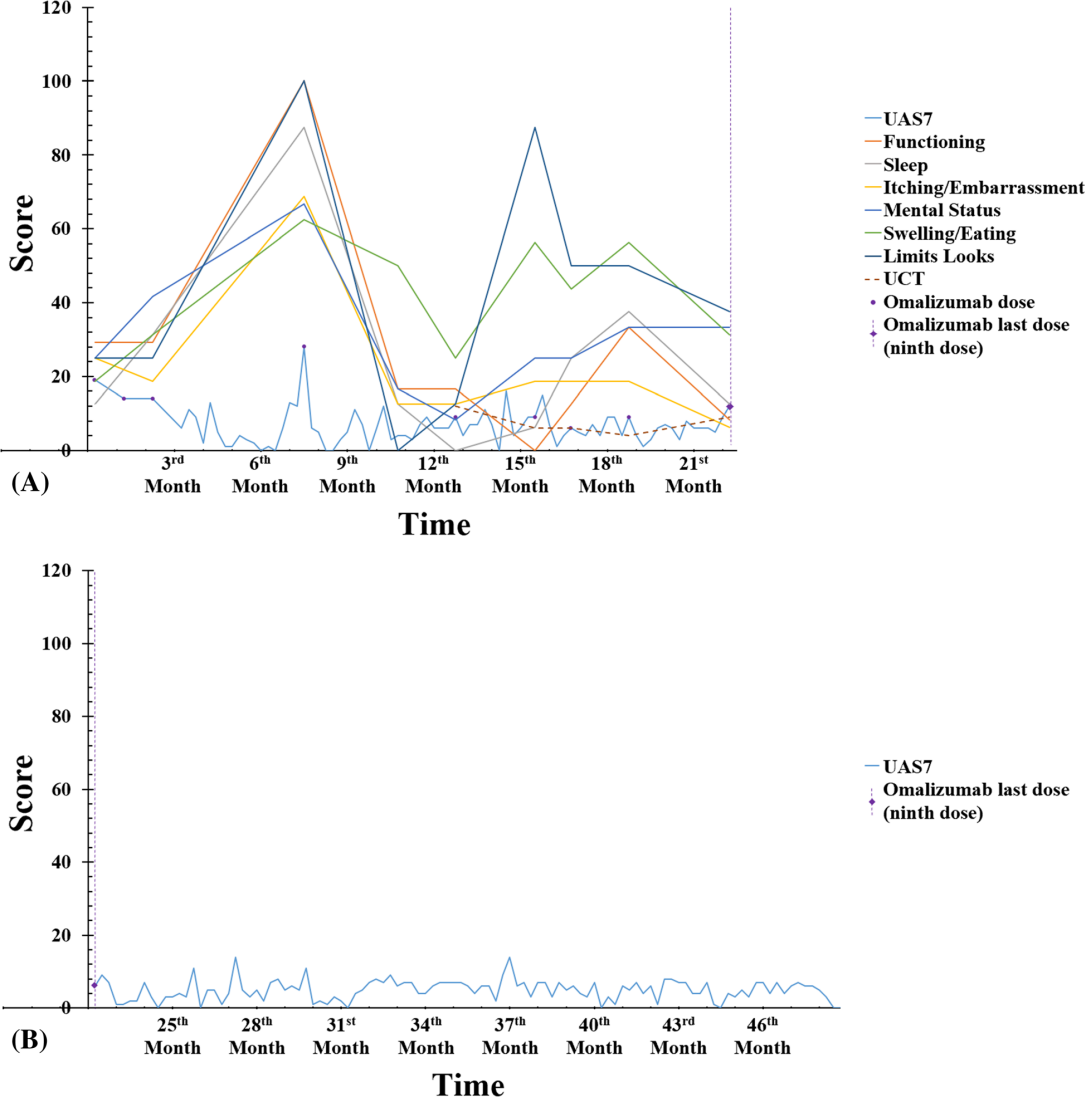
UAS7, CU-Q2oL and UCT scores over time. Data are expressed as scores in Y axis, while time is expressed in a monthly scale on X axis. (A) UAS7, CU-Q2oL and UCT scores during treatment with Omalizumab. For CU-Q2oL, each of the six domains are represented separately (functioning, sleep, itching/embarrassment, mental status, swelling/eating and limits looks). Omalizumab doses received are depicted as purple circles. (B) UAS7 scores during follow-up after treatment with Omalizumab. UAS7, Urticaria Activity Score 7; CU-Q2oL, Chronic Urticaria Quality of Life; UCT, Urticaria Control Test

Omalizumab 150 mg was administered every 4 weeks for 3 months. Second-generation antihistamines were used as needed. Both CU-Q2oL and UAS 7 improved. After three-month therapy with omalizumab, disease severity improved from moderate severity (UAS7 = 19) to well controlled (UAS7 = 6). However, due to economic limitations and insurance restrictions, the patient discontinued the treatment with omalizumab. Then, 5 months after she received her third dose, the patient complained of worsening symptoms and active disease with quality of life impairment. Thus, the fourth omalizumab (150 mg) dose was prescribed with corticosteroids. Thereafter, the patient presented a disease activity and quality of life with a fluctuating pattern that was controlled with additional doses of omalizumab (5 in total), which is depicted in Fig. 3a. While follow-up is still ongoing, the patient is currently asymptomatic without any kind of medications and her mean UAS7 score after the last administration of omalizumab (ninth dose) is 4.88, which is considered a well-controlled urticaria (Fig. 3b) [15].

## Discussion

In chronic urticaria, patient-reported outcome measures (PROMs) are important for assessing disease status and the impact of symptoms on patients’ lives [16]. However, to our knowledge, there is no validated tool to measure such outcomes in UV patients. Although UAS7 and CU-Q2oL were not designed for UV assessment, we used these tools to measure how successful or unsuccessful the first-line therapy was in managing the disease. A modest control of disease severity was considered the major criteria for prescribing omalizumab. Both questionnaires were used to observe their fluctuations over the course of the disease, serving as guides for the management and indication of omalizumab.

Understanding the role of omalizumab in UV remains controversial. Theoretically, omalizumab should improve any IgE-mediated condition, such as type I hypersensitivity reactions [17]. UV is mediated by a type III hypersensitivity reaction, with antigen-antibody complexes depositing at the vascular lamina. Omalizumab might not work in UV because its pharmacodynamics are incompatible with the pathogenesis of this disease [18].

Few publications have reported the use of omalizumab in UV. For instance, in a publication by Fueyo-Casado et al., omalizumab was initiated in a female patient with a baseline UAS7 of 32 at 300 mg monthly for 12 months. The patient achieved remission (UAS of 0) by the fifth dose. In contrast, at a baseline UAS7 of 19, we administered half the dose monthly for the first 3 months, and UAS7 improved after this time (UAS7 = 6). As the benefits of omalizumab are considered to be dose-dependent, there was some efficacy in using half of the recommended dose. Unfortunately, the omalizumab treatment was suspended due to economic limitations, as is frequently seen with many low-income patients [19, 20]. It is interesting, however, to highlight that CU-Q2oL did not improve as well as UAS7, leading to a worse quality of life even in the presence of less severe disease after the three-week treatment.

Although the correlation between UAS7 and CU-Q2oL was fairly good in the domains previously described, continuous administration with omalizumab at the beginning of treatment might be recommended, because 150 mg dose improves severity but seems insufficient to improve quality of life.

In another publication by Diez et al., a regimen of 150 mg every 4 weeks achieved remission in two patients, and Sussman et al. reported successful results in a patient who also achieved remission [21, 22]. It is unclear whether 150 mg or 300 mg should be used and for how long, but certainly quality of life should be considered in the initial assessment of UV.

Although corticosteroids are the mainstay of UV treatment, we preferred omalizumab and hydroxychloroquine because corticosteroids traditionally need to be used over the long term to prevent disease relapse, leading to more adverse effects [23]. Omalizumab and hydroxychloroquine safety profiles are better than those of corticosteroids and result in fewer complications [23]. Corticosteroids were used only during periods of highly active disease. Our patient developed an exacerbation 5 months after the three-month course of omalizumab, with the highest UAS7 (28) and the greatest impairment in quality of life. The association between UAS7 and CU-Q2oL was evident during this crisis. Both greatly improved after the administration of corticosteroids and omalizumab. This combination proved useful during exacerbations.

PROMs assist physicians in making inferences about changes in disease activity, response to treatment, and changes in health-related quality of life (HRQoL) [24]. Either the Dermatology Life Quality Index (DLQI) or CU-Q2oL might be used for chronic spontaneous urticaria (CSU) [25, 26]. However, neither have been validated for UV management. In a publication by Stull D. et al., CSU latent growth curve trajectories showed correlations between slopes of change in UAS7 and CU-Q2oL in clinical trials of ASTERIA I (0.90), ASTERIA II (0.89), and GLACIAL (0.92) [24]. Although we cannot directly compare our results due to methodological discrepancies, we also found a strong correlation between UAS7 and CU-Q2oL mean scores (*r* = .74, *p* = .022). This contrasts with another publication in which UAS7 was shown to only moderately correlate with the CU-Q2oL total score (*r* = 0.40, *p* < 0.0001) and with the DLQI score (*r* = 0.37, *p* < 0.0001) in CSU patients [27]. A weak significant correlation (*r* = 0.31, *p* < 0.05) between urticaria activity score values and DLQI in CU patients has also been reported [28].

**Fig. 4:**
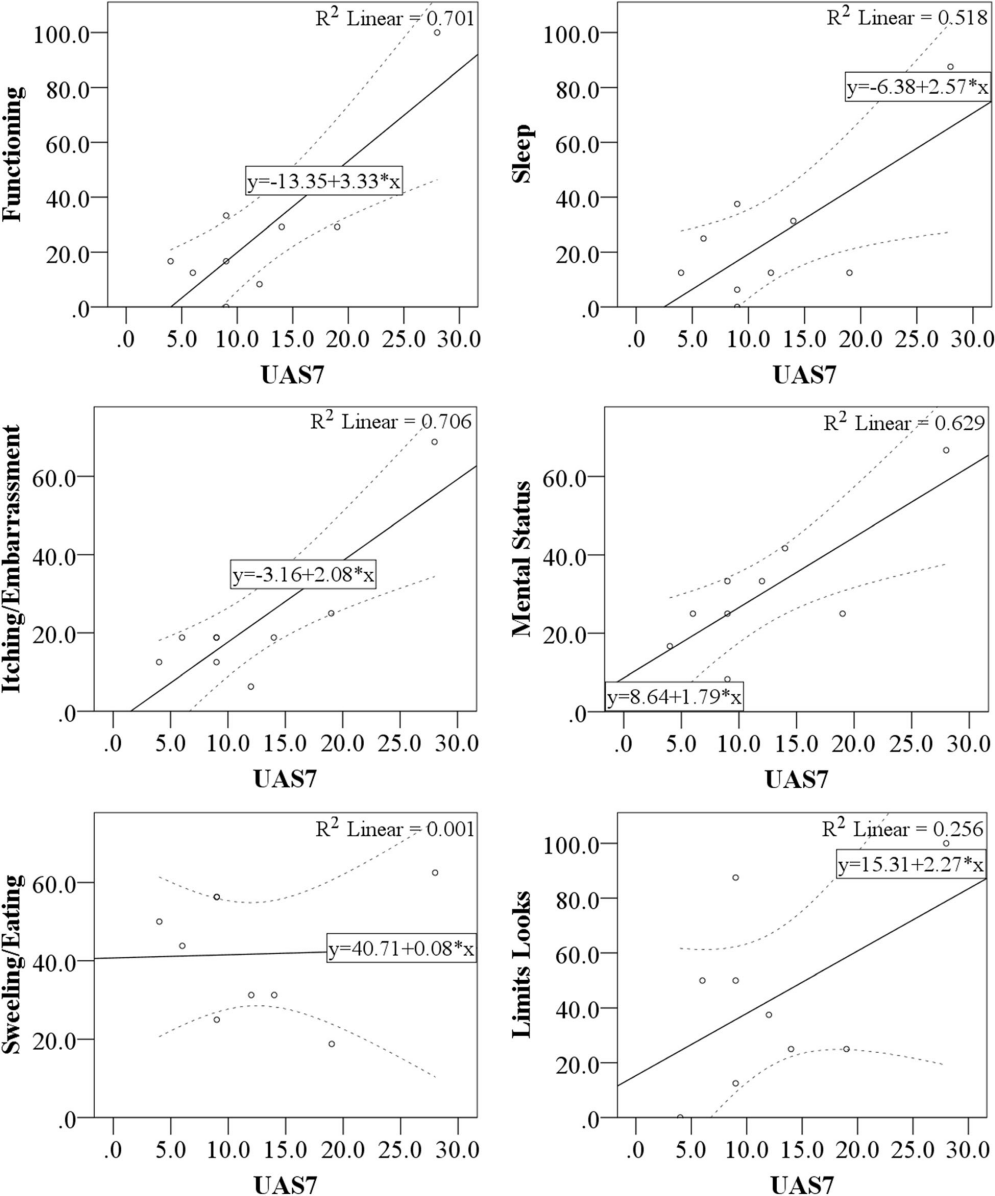
CU-Q2oL domains’ scores as a function of UAS7 scores. Each CU-Q2oL domain value in Y axis is expressed as a function of UAS7 on X axis, as depicted on each upper right box. All correlations were statistically significant, except for swelling/eating and limits looks. Continuous line represents the linear regression line of best fit, whilst dashed lines are confidence intervals (95%). UAS7, Urticaria Activity Score 7; CU-Q2oL, Chronic Urticaria Quality of Life; R^2^ linear, coefficient of determination

Interestingly, there seems to be a group of patients with relatively low UAS7 that nevertheless experience a remarkable impairment in their HRQoL [27]. This was replicated in our patient and is the reason why, in some scenarios, quality of life impairment was considered as a primary indication for omalizumab prescription independent of disease severity. Omalizumab was prescribed due to quality of life impairment on six occasions, once in the context of controlled disease and five times in mild disease assessed by UAS7. We believe that this phenomenon could be explained in part by the fact that such observation was made by analyzing quality of life as a total or average sum rather than addressing it by its axes separately as it was originally designed to be used. Certainly, discrepancy between different axes might be found. For instance, in a study of 51 CSU patients, significant correlations with UAS7 were reported for functioning (*r* = 0.31, *p* = .03), itching/embarrassment (*r* = 0.54, *p* = .001), and mental status (*r* = 0.28, *p* = .048), while those of sleep, swelling/eating, and limited appearance were not [26]. We found similar results in our patient, with stronger correlations between UAS7 and functioning (*r* = .84, *p* = .005), itching/embarrassment (*r* = .84, *p* = .005), mental status (*r* = .79, *p* = .011), and sleep (*r* = .72, *p* = .029) (Fig. 4). Correlations for swelling/eating (*r* = .04, *p* = .922) and limited appearance (*r* = .51, *p* = .165) were not statistically significant, since they did not improve as expected with treatment.

We would not recommend evaluating CU-C2oL as a single score since axis-per-axis evaluation might lead to a more thorough understanding of quality of life impairment. Under some circumstances, disruption in a single axis might be a sufficient indication to initiate omalizumab therapy even if the remainder axes have progressed favorably within a less active disease. Impairment in quality of life by itself might lead to significant morbidity. Patients with CSU often experience depression and anxiety, with studies showing a positive correlation between itch intensity and severity of depression [29]. Patients complain of recurrent pain syndromes, including tension headaches and fibromyalgia, while the prevalence of psychiatric disorders such as depression, hysteria, hypochondria, and post-traumatic stress disorder is high in these individuals [30–32].

Of note, to prevent such comorbidities, it is essential that every prescribed therapeutic regimen be evaluated objectively with parameters of disease control. Although it is not validated for urticarial vasculitis, the Spanish version of UCT was discretely used since month 13 [33]. UCT is a validated instrument designed to assess the level of disease control in CU patients, in which a score ≥ 12 indicates well-controlled urticaria, while a score of ≤11 indicates poor disease control [34]. UCT has been shown to correlate slightly better with the total CU-Q2oL score than with UAS7 [34]. The mean score of the five UCT questionnaires answered by our patient revealed poor control. In general, UCT scores did not correlate with either UAS7 or each of the CU-Q2oL domains. However, UCT correlated strongly with the total CU-Q2oL score (*r* = −.95, *p* = .023). Although we have very limited data from this test, it was the least useful method for managing the disease.

Little is known about the role of omalizumab in UV and how treatment should be followed-up in terms of disease activity and PROMs improvement. Based on our observations, we believe omalizumab 150 mg might be a feasible therapeutic alternative when first-line treatment is unsuccessful. Omalizumab in UV has shown improvement in quality of life and disease severity without the significant adverse effects caused by first-line regimens. UAS7 and CU-Q2oL might be useful in the clinical setting as objective measures to determine treatment efficacy. However, some domains in the CU-Q2oL questionnaires do not correlate well with UAS7, which might serve as a relative indication to continue treatment despite disease severity improvement. Further studies are needed to confirm our findings.

## Abbreviations

AZA: Azathioprine
CU: Chronic urticaria
CU-Q2oL: Chronic Urticaria Quality of Life Questionnaire
DLQI: Dermatology Life Quality Index
HQC: Hydroxychloroquine
HRQoL: Health-related quality of life
LCV: Leukocytoclastic vasculitis
MMF: Mycophenolate mofetil
PROMs: Patient-reported outcome measures
SLE: Systemic lupus erythematosus
UAS7: Urticaria activity score
UCT: Urticaria control test
UV: Urticarial vasculitis
